# Supplementary material for: Improved reconstruction of single-cell developmental potential with CytoTRACE 2
Source: Nat Methods. 2025 Oct 27;22(11):2258–63. doi: 10.1038/s41592-025-02857-2 (PMC12615260; doi:10.1038/s41592-025-02857-2)
Supplement: Supplementary file 1 — Supplementary Note. [file 41592_2025_2857_MOESM1_ESM.pdf]

nature methods

Supplementary information https://doi.org/10.1038/s41592-025-02857-2

Improved reconstruction of single-cell developmental potential with CytoTRACE 2

In the format provided by the authors and unedited

Supplementary Note

Impact of technical factors on CytoTRACE 2 performance

To evaluate robustness to annotation errors, we applied a noisy labeling strategy[1], showing that predictions remain stable even when up to 20% of cells or phenotypes were misannotated in training (**Extended Data Fig. 3a-c**). We also tested the limits of CytoTRACE 2's performance under varying mRNA content and data sparsity. Predictions remained consistent with as few as 750 detectably expressed genes per cell (**Extended Data Fig. 3d**), 2,000 unique molecular identifiers per cell (**Extended Data Fig. 3e**), and ~5 cells per phenotype (**Extended Data Fig. 3f**). These findings demonstrate that CytoTRACE 2 is resistant to moderate annotation errors and performs reliably under practical data limitations.

Potency annotation scheme

We used the following annotation scheme to assign single-cell transcriptomes from our potency atlas to six broad potency categories[2-6]. Where possible, we also annotated developmental orderings within each broad potency category, resulting in a larger repertoire of 24 granular potency levels spanning the full range of cellular ontogeny (n = 2 in 'Totipotent', n = 6 in 'Pluripotent', n = 7 in 'Multipotent', n = 4 in 'Oligopotent', n = 3 in 'Unipotent', n = 2 in 'Differentiated'; **Fig. 1b**; **Supplementary Tables 2** and **3**). However, such finer-grained orderings have sparse representation across species and scRNA-seq datasets; therefore, we restricted model training to the six broader categories. Rationale for the 24 granular states and citations supporting the classification of every cell type with assignable potency in this study are provided in **Supplementary Tables 2** and **3**. Granular potency levels are also indicated in the descriptions below as numeric values between "1" (highest potency) and "24" (lowest potency).

Totipotent. Totipotency was defined as the potential of a cell to give rise to all the cell types in a body, including extraembryonic cells, with maternal support. In addition to the totipotent zygote ("1"), we included the 2-cell stage of mouse and human embryos in which initial zygotic division has formed a 2-cell embryo ("2"). This assignment was supported by experiments in mammalian models showing development of physiologically normal organisms after re-implantation of zygote and blastomeres from the 2-cell stage[7-9].

Pluripotent. Pluripotency was defined as the potential of a cell to give rise to all the cell types in a body except extraembryonic cells. Although there are some reports that suggest blastomeres from the 4-cell stage are totipotent[10], given mixed reports and uncertainty in humans, we conservatively assigned the 4-cell stage as the most primitive cell state in the pluripotent category ("3"). Chronologically, the 8-cell stage ("4") and 16-cell stage ("5") – referred to as a morula – come next. The embryo then progresses to the 32-cell stage ("6") when it undergoes compaction, in which a central cavity forms and cells separate to give rise to the trophectoderm and inner cell mass, the latter of which is the origin of embryonic stem cells[11]. As the pluripotent cells divide and specialize, they transition from the early blastocyst through the mid- ("7") and late- ("8") blastocyst stages, which mark the final stages of pluripotency in our analysis.

Multipotent. Multipotency was defined as the potential of an immature cell to give rise to multiple cell types (at least four) across different lineages. Multipotent cells exist both during early embryogenesis for specification of germ layers (mesoderm, endoderm, ectoderm) and during the postnatal period as tissue-resident stem cell populations. Chronologically, the anterior primitive streak is the earliest source of multipotent cells ("9"), which sequentially gives rise to developmentally restricted cells in each germ layer (e.g., paraxial mesoderm ("10"), which then

segments into somitomeres (“11”) and somites (“12”))[12]. Development occurs in parallel across germ layers, so the granular order of multilineage precursors from different lineages was aligned to relative developmental time using author-provided labels for embryonic age or somite stage. For example, pre-cranial neural crest cells (pre-CNCCs; “12”) arise during the mouse 4-somite stage[13], so their developmental timeline matched early somites (“12”). These cells then give rise to CNCCs (“13”) and early multipotent precursors (“14”), such as the delaminating CNCC, dermomyotome, and sclerotome. Given their tissue-specific developmental potency, tissue-resident multipotent stem and progenitor cells, such as hematopoietic stem and progenitors, pancreatic progenitors, skeletal stem cells, radial glial cells, and others, were annotated as the least primitive among multipotent cells (“15”).

Oligopotent. Oligopotency was defined as the potential of an immature cell to give rise to more than one but fewer than four cell types across different lineages and/or cell types immediately downstream of known multipotent stem and progenitor cells. In our potency atlas, the most primitive cells in this category are unpurified hematopoietic progenitors, such as $CD34^+$ cells, which are enriched for restricted progenitors but also contain rare multipotent hematopoietic stem cells (“16”). Sequentially, cells were granularly ordered as oligopotent progenitor populations without rare multipotent cells (“17”)[14-16], restricted bipotent stem and progenitor cells (“18”)[17,18], and then basal cells (“19”)[19-24] which are a heterogeneous mixture of predominantly bipotent progenitors, along with unipotent progenitors, differentiated cells, and rare multipotent cells capable of regeneration in response to injury.

Unipotent. Unipotency was defined as the potential of an immature cell to predominantly give rise to one mature downstream cell type. In our potency atlas, the most primitive cells in this category are unipotent stem-like cells, such as limbal stem cells[25,26], satellite stem cells[27], and nascent type II pneumocytes (“20”). Sequentially, cells were granularly ordered as unipotent progenitors without self-renewal capacity (“21”), followed by immature cells transitioning toward differentiation (“22”)[28].

Differentiated. Differentiated cells were defined as mature cells within a single lineage, including non-terminally differentiated cells (“23”), which have limited potential to further differentiate or change phenotypic state in response to a stimulus (e.g., naïve B cells and naïve T cells upon antigen recognition; fibroblasts and other stromal cells in response to tissue injury), and terminally differentiated cells (“24”), which essentially have zero potential to make other cells, including of their same type.

Additional details, including lineage contributions, and references supporting these classifications are provided in **Supplementary Tables 2** and **3**.

Supplementary Note References

1. Natarajan, N., Dhillon, I.S., Ravikumar, P. & Tewari, A. Learning with noisy labels. in *Proceedings of the 27th International Conference on Neural Information Processing Systems - Volume 1*, Vol. 1 1196–1204 (Curran Associates Inc., Lake Tahoe, Nevada, 2013).
2. Kolios, G. & Moodley, Y. Introduction to Stem Cells and Regenerative Medicine. *Respiration* **85**, 3-10 (2012).
3. Gabr, H.M. & El-Kheir, W.A. Chapter 3 - Stem cells: definition, biological types, classifications, and properties. in *Stem Cell Therapy* (eds. Gabr, H.M. & El-Kheir, W.A.) 21-33 (Academic Press, 2023).
4. Zomer, H.D., Vidane, A.S., Gonçalves, N.N. & Ambrósio, C.E. Mesenchymal and induced pluripotent stem cells: general insights and clinical perspectives. *Stem Cells Cloning* **8**, 125-134 (2015).
5. De Los Angeles, A.*, et al.* Hallmarks of pluripotency. *Nature* **525**, 469-478 (2015).
6. Wagers, A.J. & Weissman, I.L. Plasticity of Adult Stem Cells. *Cell* **116**, 639-648 (2004).
7. Tarkowski, A.K. Experiments on the Development of Isolated Blastomeres of Mouse Eggs. *Nature* **184**, 1286-1287 (1959).
8. Allen, W.R. & Pashen, R.L. Production of monozygotic (identical) horse twins by embryo micromanipulation. *J Reprod Fertil* **71**, 607-613 (1984).
9. Matsumoto, K., Miyake, M., Utsumi, K. & Iritani, A. Production of identical twins by separating two-cell rat embryos. *Gamete Res* **22**, 257-263 (1989).
10. Maemura, M.*, et al.* Totipotency of mouse zygotes extends to single blastomeres of embryos at the four-cell stage. *Sci Rep* **11**, 11167 (2021).
11. Schoenwolf, G.C. & Larsen, W.J. *Larsen's human embryology*, (Churchill Livingstone/Elsevier, Philadelphia, 2009).
12. Loh, K.M.*, et al.* Mapping the Pairwise Choices Leading from Pluripotency to Human Bone, Heart, and Other Mesoderm Cell Types. *Cell* **166**, 451-467 (2016).
13. Zalc, A.*, et al.* Reactivation of the pluripotency program precedes formation of the cranial neural crest. *Science* **371**(2021).
14. Ranzoni, A.M.*, et al.* Integrative Single-Cell RNA-Seq and ATAC-Seq Analysis of Human Developmental Hematopoiesis. *Cell Stem Cell* **28**, 472-487 e477 (2021).
15. Lo Giudice, Q., Leleu, M., La Manno, G. & Fabre, P.J. Single-cell transcriptional logic of cell-fate specification and axon guidance in early-born retinal neurons. *Development* **146**(2019).
16. Bastidas-Ponce, A.*, et al.* Comprehensive single cell mRNA profiling reveals a detailed roadmap for pancreatic endocrinogenesis. *Development* **146**(2019).
17. Desai, T.J., Brownfield, D.G. & Krasnow, M.A. Alveolar progenitor and stem cells in lung development, renewal and cancer. *Nature* **507**, 190-194 (2014).
18. Fogg, D.K.*, et al.* A clonogenic bone marrow progenitor specific for macrophages and dendritic cells. *Science* **311**, 83-87 (2006).
19. Visvader, J.E. & Stingl, J. Mammary stem cells and the differentiation hierarchy: current status and perspectives. *Genes Dev* **28**, 1143-1158 (2014).
20. Okubo, T., Clark, C. & Hogan, B.L. Cell lineage mapping of taste bud cells and keratinocytes in the mouse tongue and soft palate. *Stem Cells* **27**, 442-450 (2009).
21. Blanpain, C. & Fuchs, E. Epidermal stem cells of the skin. *Annu Rev Cell Dev Biol* **22**, 339-373 (2006).
22. Rawlins, E.L. & Hogan, B.L. Epithelial stem cells of the lung: privileged few or opportunities for many? *Development* **133**, 2455-2465 (2006).
23. Goldstein, A.S.*, et al.* Purification and direct transformation of epithelial progenitor cells from primary human prostate. *Nat Protoc* **6**, 656-667 (2011).

24. Garraway, I.P., *et al.* Human prostate sphere-forming cells represent a subset of basal epithelial cells capable of glandular regeneration in vivo. *Prostate* **70**, 491-501 (2010).
25. Gonzalez, G., Sasamoto, Y., Ksander, B.R., Frank, M.H. & Frank, N.Y. Limbal stem cells: identity, developmental origin, and therapeutic potential. *Wiley Interdiscip Rev Dev Biol* **7**(2018).
26. Cotsarelis, G., Cheng, S.Z., Dong, G., Sun, T.T. & Lavker, R.M. Existence of slow-cycling limbal epithelial basal cells that can be preferentially stimulated to proliferate: implications on epithelial stem cells. *Cell* **57**, 201-209 (1989).
27. Brack, A.S. & Rando, T.A. Tissue-specific stem cells: lessons from the skeletal muscle satellite cell. *Cell Stem Cell* **10**, 504-514 (2012).
28. Barkauskas, C.E., *et al.* Type 2 alveolar cells are stem cells in adult lung. *J Clin Invest* **123**, 3025-3036 (2013).
