# Supplementary material for: Improved reconstruction of single-cell developmental potential with CytoTRACE 2
Source: Nat Methods. 2025 Oct 27;22(11):2258–63. doi: 10.1038/s41592-025-02857-2 (PMC12615260; doi:10.1038/s41592-025-02857-2)
Supplement: Supplementary file 2 — Reporting Summary [file 41592_2025_2857_MOESM2_ESM.pdf]

nature portfolio

Corresponding author(s): Aaron M. Newman

Last updated by author(s): 6/13/2025

Reporting Summary

Nature Portfolio wishes to improve the reproducibility of the work that we publish. This form provides structure for consistency and transparency in reporting. For further information on Nature Portfolio policies, see our Editorial Policies and the Editorial Policy Checklist.

Statistics

For all statistical analyses, confirm that the following items are present in the figure legend, table legend, main text, or Methods section.

n/a	Confirmed	
☐	☒	The exact sample size (*n*) for each experimental group/condition, given as a discrete number and unit of measurement
☐	☒	A statement on whether measurements were taken from distinct samples or whether the same sample was measured repeatedly
☐	☒	The statistical test(s) used AND whether they are one- or two-sided<br>*Only common tests should be described solely by name; describe more complex techniques in the Methods section.*
☐	☒	A description of all covariates tested
☐	☒	A description of any assumptions or corrections, such as tests of normality and adjustment for multiple comparisons
☐	☒	A full description of the statistical parameters including central tendency (e.g. means) or other basic estimates (e.g. regression coefficient) AND variation (e.g. standard deviation) or associated estimates of uncertainty (e.g. confidence intervals)
☐	☒	For null hypothesis testing, the test statistic (e.g. *F*, *t*, *r*) with confidence intervals, effect sizes, degrees of freedom and *P* value noted<br>*Give P values as exact values whenever suitable.*
☒	☐	For Bayesian analysis, information on the choice of priors and Markov chain Monte Carlo settings
☐	☒	For hierarchical and complex designs, identification of the appropriate level for tests and full reporting of outcomes
☐	☒	Estimates of effect sizes (e.g. Cohen's *d*, Pearson's *r*), indicating how they were calculated

Our web collection on statistics for biologists contains articles on many of the points above.

Software and code

Policy information about availability of computer code

Data collection	Standard FACSDiva software (v9.7) was used for flow cytometry on a BD FACSAria II. Fluorescence images were acquired on a Zeiss LSM 980 confocal microscope. Publicly available FASTQ files were downloaded using sra-tools v3.1.1.
Data analysis	Software packages used in this study are detailed in Methods, including CytoTRACE 1 v0.3.3, scPred v1.9.2, pySingleCellNet v0.1.1, scmap v1.26.0, scikit-learn v1.1.1 & v1.4.2, XGBoost v2.1.1, SCENT v1.0.3, FitDevo v1.2.0, SLICE v0.99.0, RaceID v0.1.4, scTour v1.0.0, org.Hs.eg.db v3.15.0, cutadapt v4.9, dropest v0.8.6, STAR v2.7.11b, Cell Ranger v8.0.1, velocyto.py v0.17.17, scVelo v0.3.1, and wandb v0.16.4. Seurat versions 4.3.0 and 5.1.0, fgsea v1.25.1, GSVA v1.46.0, RANN v2.6.1, HiClimR v2.2.1, and various R v4.2+ packages (e.g., ggplot2 v3.4.3, matrix v1.6.1, dplyr 1.1.3) and python v3.9+ packages (e.g., pandas v2.2.3, numpy v1.26.3) were also used. CytoTRACE 2 results were generated with version 1.1.0.3 (cytotrace2-py) which uses python v3.9.0 and PyTorch v2.0.0.<br><br>Flow cytometry data was analyzed with FlowJo (v10.9.0). Fluorescence images were analyzed with ImageJ (v1.53t) to obtain mean fluorescence intensities.

For manuscripts utilizing custom algorithms or software that are central to the research but not yet described in published literature, software must be made available to editors and reviewers. We strongly encourage code deposition in a community repository (e.g. GitHub). See the Nature Portfolio guidelines for submitting code & software for further information.

Data

Policy information about availability of data

All manuscripts must include a data availability statement. This statement should provide the following information, where applicable:

- Accession codes, unique identifiers, or web links for publicly available datasets
- A description of any restrictions on data availability
- For clinical datasets or third party data, please ensure that the statement adheres to our policy

All datasets comprising the single-cell potency atlas assembled in this work (Supplementary Table 1) are publicly available from the Gene Expression Omnibus (GEO), ArrayExpress, or the Sequence Reach Archive (SRA) with the following accessions: GSE52583 ('AT2/AT1 lineage (C1)'), GSE109774 ('Bone marrow (10x)', 'Bone marrow (Smart-seq2)', and 'Tabula Muris (Smart-seq2/10x)'), GSE60783 ('Dendritic cells (C1)'), GSE97391 ('Direct in vitro neuron (inDrop)' and 'Standard in vitro neuron (inDrop)'), GSE70245 ('HSPCs (C1)'), GSE113197 ('Human breast 1 (10x)' and 'Human breast 1 (C1)'), GSE161529 ('Human breast 2 (10x)'), GSE36552 ('Human embryo (Tang et al.)'), GSE92332 ('Intestine (Drop-seq)' and 'Intestine (Smart-seq2)'), GSE85066 ('Mesoderm (C1)'), GSE45719 ('Mouse embryo 1 (Tang et al.)'), SRP073767 ('Peripheral blood (10x)'), GSE128639 ('BM-MNC (CITE-seq)'), GSE100866 ('Cord blood (CITE-seq)'), E-MTAB-9067 ('HSC development (Smart-seq2)'), GSE90742 ('HSCs and MPPs (inDrop)'), E-MTAB-11536 ('Immune cell atlas (10x)'), GSE76408 ('Lgr5-CreER intestine (CEL-seq)'), E-MTAB-3321 ('Mouse embryo 2 (Smart-seq2)'), GSE59892 ('Mouse embryo 3 (Smart-seq)'), GSE162044 ('Neural crest (Smart-seq2)'), GSE132188 ('Pancreas (10x)'), GSE99933 ('Peripheral glia (Smart-seq2)'), GSE122466 ('Retinal neurons (10x)'), GSE64447 ('Skeletal stem cell (C1)'), and GSE201333 ('Tabula Sapiens (Smart-seq2/10x)').

Raw FASTQ or BAM files analyzed in this work are available from the SRA with the following accessions: SRP188993 ('BM-MNC (CITE-seq)'), SRP168426 ('Retinal neurons (10x)'), SRP200419 ('Pancreas (10x)'), SRP109011 ('Peripheral glia (Smart-seq2)'), SRP239468 ('Skeletal stem cell (C1)'), SRP094420 ('HSCs and MPPs (inDrop)'), and SRP476153 ('Mouse neurogenesis (10x)' and 'Mouse mature neural cell types (10x)').

Five expression datasets covering mouse embryogenesis periods from implantation to organogenesis are accessible from GEO or ArrayExpress with the following accessions: GSE100597 ('Implantation (E3.5-E6.5)'), GSE109071 ('Implantation (E5.5-E6.5)'), E-MTAB-6967 ('Gastrulation (E6.5-E8.5)'), GSE186069 ('Organogenesis (E8.5)'), and GSE228590 ('Organogenesis (E8.75-P0)').

The publicly available oligodendroglioma and AML expression data analyzed in this work are available with GEO accession numbers GSE70630 and GSE116256, respectively.

Reference genomes and annotation files for GRCm39 (mouse) and GRCh38.p13 (human) were obtained from Ensembl release 109 (February 2023) via the archive at https://feb2023.archive.ensembl.org.

Research involving human participants, their data, or biological material

Policy information about studies with human participants or human data. See also policy information about sex, gender (identity/presentation), and sexual orientation and race, ethnicity and racism.

Reporting on sex and gender	No human data was generated for this study.
Reporting on race, ethnicity, or other socially relevant groupings	N/A
Population characteristics	N/A
Recruitment	N/A
Ethics oversight	N/A

Note that full information on the approval of the study protocol must also be provided in the manuscript.

Field-specific reporting

Please select the one below that is the best fit for your research. If you are not sure, read the appropriate sections before making your selection.

☒ Life sciences ☐ Behavioural & social sciences ☐ Ecological, evolutionary & environmental sciences

For a reference copy of the document with all sections, see nature.com/documents/nr-reporting-summary-flat.pdf

Life sciences study design

All studies must disclose on these points even when the disclosure is negative.

Sample size	For both single-cell RNA-seq studies and mouse experiments, sample sizes were based on prior studies with similar designs and optimized for feasibility. The selected sizes were sufficient to detect consistent and biologically meaningful trends across replicates and conditions, and to support the statistical analyses presented. Where applicable, findings were validated in independent cohorts or with orthogonal methods to ensure robustness. All results were analyzed and interpreted using statistically appropriate techniques as described in Methods.

Data exclusions	Quality control metrics for data exclusion are fully described in Methods. Key exclusions included scRNA-seq samples of tumors which were derived from cell lines or for which fewer than 10 malignant cells were identified, and from these samples, non-malignant cells annotated by the author as "undifferentiated". In generating the potency atlas presented in this study, phenotypes in Tabula Sapiens with fewer than five cells for a given tissue/platform pair were excluded.
Replication	The CytoTRACE 2 model was developed over a portion of the curated gold standard potency atlas, then tested over fully held-out data from the remainder as well as Tabula Sapiens data not included in either cohort. To ensure replicability and generalizability, CytoTRACE 2 was also tested in a leave-clade-out framework as described in Methods. CytoTRACE 2 performance was strongly concordant across these experiments and cohorts.<br><br>All experiments were replicated three times independently.
Randomization	To ensure generalizability and limit any bias from the primary training cohort selection, we repeated the training and testing process of CytoTRACE 2 across three additional train/test splits, generated randomly, as detailed in Methods. Performance was strongly concordant across these experiments and cohorts.<br><br>The robustness experiments in Extended Data Figure 3 were conducted with randomization and replicated five times as described in Methods. Averages across replicates were presented with confidence intervals.<br><br>For experiments with mice, randomization was not applicable as there was no treatment involved.
Blinding	The investigators were not blinded to group allocation, but the training and test cohorts analyzed in this work were generated without prior knowledge of CytoTRACE 2 potency predictions. The randomization framework detailed above serves as an additional control.

Reporting for specific materials, systems and methods

We require information from authors about some types of materials, experimental systems and methods used in many studies. Here, indicate whether each material, system or method listed is relevant to your study. If you are not sure if a list item applies to your research, read the appropriate section before selecting a response.

Materials & experimental systems

n/a	Involved in the study	
☐	☒	Antibodies
☒	☐	Eukaryotic cell lines
☒	☐	Palaeontology and archaeology
☐	☒	Animals and other organisms
☒	☐	Clinical data
☒	☐	Dual use research of concern
☒	☐	Plants

Methods

n/a	Involved in the study	
☒	☐	ChIP-seq
☐	☒	Flow cytometry
☒	☐	MRI-based neuroimaging

Antibodies

Antibodies used	Immunostaining antibody:<br>anti-E-Cadherin-Alexa Fluor 488 antibody (BD Biosciences 560061, 1:50)<br><br>Flow cytometry antibodies:<br>anti-mouse lineage cocktail-A700 (BioLegend 133313, 5 μl per mouse)<br>anti-CD117 (c-Kit)-BV395 (Thermo Fisher Scientific 363-1171-80, 1:100)<br>anti-Sca1-BV605 (BioLegend 108133, 1:100)<br>anti-CD34-eFluor 450 (Thermo Fisher Scientific 48-0341-80, 1:40),<br>anti-CD16/32-BV711 (BD Biosciences 740659, 1:100)<br>anti-CD135-BV421 (BioLegend 135313, 1:100)<br>anti-CD127 (IL-7Rα)-BV711 (BioLegend 135035, 1:100)<br>anti-CD3-BV711 (BioLegend 100241, 1:100)<br>anti-CD8a-BV605 (BioLegend 100743, 1:100)<br>anti-CD19-BV605 (BioLegend 115539, 1:100)
Validation	All antibodies used were validated by the respective manufactures. The validation statement of the antibodies on the manufacture's website can be found below.<br>anti-E-Cadherin-Alexa Fluor 488 antibody (https://www.bdbiosciences.com/en-us/products/reagents/microscopy-imaging-reagents/immunofluorescence-reagents/alexa-fluor-488-mouse-anti-e-cadherin.560061?tab=product_details), anti-mouse lineage cocktail-A700 (https://www.biolegend.com/en-us/products/alexa-fluor-700-anti-mouse-lineage-cocktail-with-isotype-ctrl-8122), anti-CD117 (c-Kit)-BV395 (https://www.thermofisher.com/antibody/product/CD117-c-Kit-Antibody-clone-2B8-Monoclonal/363-1171-80), anti-Sca1-BV605 (https://www.biolegend.com/en-us/products/brilliant-violet-605-anti-mouse-ly-6a-e-sca-1-antibody-8664), anti-CD34-eFluor 450 (https://www.thermofisher.com/antibody/product/CD34-Antibody-clone-RAM34-Monoclonal/48-0341-80), anti-CD16/32-BV711 (https://www.bdbiosciences.com/en-us/products/reagents/flow-cytometry-reagents/research-reagents/single-color-antibodies-ruo/bv711-rat-anti-mouse-cd16-cd32.740659?tab=product_details), anti-CD135-BV421 (https://www.biolegend.com/en-us/products/brilliant-violet-421-anti-mouse-cd135-antibody-8728), anti-CD127 (IL-7Rα)-BV711 (https://

www.biolegend.com/en-us/products/brilliant-violet-711-anti-mouse-cd127-il-7ralpha-antibody-10632), anti-CD3-BV711 (https://www.biolegend.com/en-us/products/brilliant-violet-711-anti-mouse-cd3-antibody-10022), anti-CD8a-BV605 (https://www.biolegend.com/en-us/products/brilliant-violet-605-anti-mouse-cd8a-antibody-7636), and anti-CD19-BV605 (https://www.biolegend.com/en-us/products/brilliant-violet-605-anti-mouse-cd19-antibody-7645)

Animals and other research organisms

Policy information about studies involving animals; ARRIVE guidelines recommended for reporting animal research, and Sex and Gender in Research

Laboratory animals	8- to 12-week-old C57BL/6 mice were used.
Wild animals	The study did not involve wild animals.
Reporting on sex	Equal numbers of males and females were used.
Field-collected samples	This study did not involve samples collected from the field.
Ethics oversight	All animal procedures were conducted according to a protocol approved by the Stanford University APLAC committee (10868).

Note that full information on the approval of the study protocol must also be provided in the manuscript.

Plants

Seed stocks	*Report on the source of all seed stocks or other plant material used. If applicable, state the seed stock centre and catalogue number. If plant specimens were collected from the field, describe the collection location, date and sampling procedures.*
Novel plant genotypes	*Describe the methods by which all novel plant genotypes were produced. This includes those generated by transgenic approaches, gene editing, chemical/radiation-based mutagenesis and hybridization. For transgenic lines, describe the transformation method, the number of independent lines analyzed and the generation upon which experiments were performed. For gene-edited lines, describe the editor used, the endogenous sequence targeted for editing, the targeting guide RNA sequence (if applicable) and how the editor was applied.*
Authentication	*Describe any authentication procedures for each seed stock used or novel genotype generated. Describe any experiments used to assess the effect of a mutation and, where applicable, how potential secondary effects (e.g. second site T-DNA insertions, mosiacism, off-target gene editing) were examined.*

Flow Cytometry

Plots

Confirm that:

☒ The axis labels state the marker and fluorochrome used (e.g. CD4-FITC).

☒ The axis scales are clearly visible. Include numbers along axes only for bottom left plot of group (a 'group' is an analysis of identical markers).

☒ All plots are contour plots with outliers or pseudocolor plots.

☒ A numerical value for number of cells or percentage (with statistics) is provided.

Methodology

Sample preparation	Hips, femurs, tibia, and humeri were harvested from C57BL/6 mice. Bones were cleaned, cut, and flushed with a syringe filled with ice-cold FACS buffer (2% fetal bovine serum [FBS] in Hanks' Balanced Salt Solution [HBSS] buffer). Cells in FACS buffer were filtered through a 40 µm filter, pelleted, and then incubated in ammonium-chloride-potassium (ACK) lysis buffer for 5 minutes on ice. Cells were then spun down and resuspended in 400 µl FACS buffer per mouse. Lineage depletion beads (Miltenyi Biotec 130-110-470) were added to the cells (50 µl per mouse) and incubated for 10 min at 4°C. After incubation, the cells were loaded onto an LS magnetic separation column (Miltenyi Biotec 130-042-401), which was subsequently washed with 3 × 3 mL of FACS buffer. Before and after washing, pass-through cells were collected, spun down, and resuspended in FACS buffer.<br><br>Blood samples were collected from the same mice for the isolation of CD8a+ T cells (CD3+ CD8a+) and B (CD19+) cells. Peripheral blood mononuclear cell (PBMC) isolation was performed using a SepMate™-15 tube (STEMCELL technologies 85415) according to the manufacturer's instructions.
Instrument	The cells were analyzed on a BD FACSAria II sorter.
Software	Data were analyzed with FlowJo V10.

Cell population abundance	The post-sort samples were re-analyzed using FACS to verify a purity level of over 95%.
Gating strategy	The major cell populations were first identified within the FSC/SSC plots, followed by doublet exclusion. The mouse HSC, MPP, T cell, and B cell populations were then gated according to previously published gating strategies (PMID: 33236985). Fluorescence-minus-one (FMO) controls were used to discriminate between positive and negative staining.

☒ Tick this box to confirm that a figure exemplifying the gating strategy is provided in the Supplementary Information.
